# Overexpression of the *AGL42* gene in cotton delayed leaf senescence through downregulation of NAC transcription factors

**DOI:** 10.1038/s41598-022-25640-1

**Published:** 2022-12-06

**Authors:** Ayesha Latif, Saira Azam, Naila Shahid, Muhammad R. Javed, Zeshan Haider, Aneela Yasmeen, Sahar Sadaqat, Mohsin Shad, Tayyab Husnain, Abdul Q. Rao

**Affiliations:** 1 Centre of Excellence in Molecular Biology (CEMB), University of the Punjab, Lahore, Pakistan; 2 Department of Bioinformatics and Biotechnology, Government College University Faisalabad, (GCUF), Allama Iqbal Road, Faisalabad, 38000 Pakistan

**Keywords:** Biotechnology, Molecular biology

## Abstract

Premature leaf senescence negatively influences the physiology and yield of cotton plants. The conserved IDLNL sequence in the C-terminal region of AGL42 MADS-box determines its repressor potential for the down regulation of senescence-related genes. To determine the delay in premature leaf senescence, Arabidopsis *AGL42* gene was overexpressed in cotton plants. The absolute quantification of transgenic cotton plants revealed higher mRNA expression of *AGL42* compared to that of the non-transgenic control. The spatial expression of GUS fused with *AGL42* and the mRNA level was highest in the petals, abscission zone (flower and bud), 8 days post anthesis (DPA) fiber, fresh mature leaves, and senescenced leaves. The mRNA levels of different NAC senescence-promoting genes were significantly downregulated in AGL42 transgenic cotton lines than those in the non-transgenic control. The photosynthetic rate and chlorophyll content were higher in AGL42 transgenic cotton lines than those in the non-transgenic control. Fluorescence in situ hybridization of the AG3 transgenic cotton line revealed a fluorescent signal on chromosome 1 in the hemizygous form. Moreover, the average number of bolls in the transgenic cotton lines was significantly higher than that in the non-transgenic control because of the higher retention of floral buds and squares, which has the potential to improve cotton fiber yield.

## Introduction

Plant aging causes senescence—the natural termination process of leaves. Leaf senescence is a highly intricate and automated process that is closely associated with the deterioration of chlorophyll levels, disruption of nucleic acids and proteins, and a decline in photosynthetic rate^1^. Several transcription factors (TFs), including bZIP, NAC, AP2/EREBP, MYB, and WRKY, are upregulated during leaf senescence^2^.

Cotton (Gossypium spp.), the most important source of fiber in the textile industry, is a major regulator of economic growth in several agriculture-based countries^3^. However, global climatic change and various biotic and abiotic factors, including light, temperature, water stress, insects, and inorganic nutrients, are responsible for premature leaf shedding, which negatively affects overall cotton yield and fiber quality^4^. Moreover, the environmental impact on cotton leaves and boll shedding appears mainly in response to hormonal imbalances such as abscisic acid (ABA), ethylene, silicic acid, and jasmonic acid^5^. Cotton contains NAC domain TFs that are highly involved in leaf senescence. Ten *GhNAC* genes (*GhNAC8–GhNAC17*) were found to be differentially expressed during natural and induced leaf senescence. Quantitative reverse transcription-polymerase chain reaction (qRT-PCR) analysis revealed the upregulation of these GhNAC genes in senesced leaves under ABA, ethylene, drought, salinity, cold, heat, and other hormonal stresses, except for *GhNAC10* and *GhNAC13*, which have maximum expression at 25 DPA fibers^6^. Moreover, overexpression of *GhNAC12* is associated with premature senescence in Arabidopsis^7^. A novel *GhNAP* gene subfamily of NAC TFs has been isolated from upland cotton, showing the highest response to leaf senescence by regulating ABA pathways. Downregulation of *GhNAP* delayed premature cotton senescence, and its overexpression led to earlier senescence in Arabidopsis. Therefore, suppression of certain NAC genes delays premature leaf senescence, thereby indirectly improving cotton yield and cotton fiber quality^8^.

The acronym MADS originates from MCM1 in yeast, AGAMOUS (AG) in Arabidopsis, DEFICIENS in Antirrhinum, and SRF in humans, which are the founding members of the largest and diverse group of TFs known for their involvement in the regulation of various developmental and signal transduction pathways in plants^9^. Some of these MADS-box DNA segments are responsible for repressing targeted gene expression^10^. Ectopic expression-mediated phenotypes suggest that the MADS-box protein *AGL15* is a component of the signaling pathway that is crucial for maintaining immature or young tissues in plants^11^. *AGL15* is highly expressed as a positive and negative regulator of somatic embryogenesis and seed development^12^. Similar phenotypes in plants overexpress *AGL18*^13^. Both TFs have similar consensus motifs ((L/F) DLN (L/F) XP) in their ERF-associated amphiphilic repressor (EAR) domains, which suppress the transcriptional activity of various zinc finger proteins that convert an activation composite into a repression complex^13^.

The constitutive expression of *AGL42* (FYF: FOREVER YOUNG FLOWER), another MADS-box protein, delays floral organ senescence and abscission in Arabidopsis by downregulating ethylene response DNA-binding factors (EDFs) and abscission-associated genes^10,14^. The presence of a conserved IDLNL sequence in the C-terminal region of the protein suggests its repressor activity owing to the similarity of this conserved sequence to the EAR motif of Class II ethylene responsive factor (ERF) repressors^15^. The optimum expression of *FYF* in young flowers before pollination is followed by a significant decline after pollination during flower development^14^. The ERF gene *FUF1* was upregulated by *FYF* during flower development. The upregulation of *FUF1* caused the same phenotype as that reported for 35S: FYF plants. The FYF-mediated delay in flower senescence is the result of increased expression of *FUF1*, which suppresses downstream genes in the ethylene response pathway EDF1/2/3/4^16^.

*IDA and BOP* are abscission-associated genes that are involved in the development of the floral abscission zone (AZ) and initiation of abscission of floral organs, respectively, and have maximum expression in the AZ. Downregulation of both *IDA* and *BOPs* in a 35S: FYF transgenic flower owing to constitutive expression of FYF (*AGL42*) before and after pollination suggests that FYF is probably expressed upstream of them. Moreover, the presence of cis-regulatory elements for MADS-box TFs in *IDA* and *BOP2* promoters further strengthens the above findings^17^. In summary, FYF-mediated suppression of *IDA* and *BOP2/1* may be responsible for the delay in senescence and abscission in 35S: FYF transgenic plants^14^. 35S: FYF Arabidopsis plants not only inhibit the abscission of flowers but also delay leaf aging. The leaf detachment assay showed that wild-type leaves suffered senescence within 3 days of detachment and dried after the 5th day. In contrast, 35S: FYF leaves remained green and non-senescent even after the 5th day of detachment^14^. A MADS-box gene, the *SIFYFL* homologue of the *FYF* gene, has been isolated from tomato and exhibits higher expression in sepal, mature leaves, different stages of fruit, and AZ. *35S: FYFL* transgenic tomatoes also displayed a delay in leaf senescence and fruit ripening, which resulted in increased storability and longer sepal length. The quantity of carotenoids also decreases in these plants. Overexpression of the *SIFYFL* gene delayed sepal and plant senescence by hindering the ethylene content, biosynthesis of ethylene, and its responsive genes compared to that of wild-type tomatoes^18^. Upregulation of the cotton MADS-box gene GhFYF, expressed in Arabidopsis, promotes salt tolerance by interacting with GhGPP2 protein and other genes, leading to increased plant growth, remarkably higher proline content, and a better rate of seed germination^19^.

Genetically engineered cotton with a delayed onset of premature senescence improves crop yield and fiber quality. The present study was planned based on the literature to overexpress the repressive protein *AGL42* (*FYF*) under a constitutive promoter in *Gossypium hirsutum* to assess its effect on the delaying of premature leaf senescence. Moreover, the responsive expression pattern of senescence *NAC* genes was investigated in transgenic cotton lines under abiotic stresses.

## Results

### Development of 35S_AGL42 transgenic cotton lines

The ligated *AGL42* gene in pCAMBIA 1301 was screened using PCR. The amplified product of 506 bp confirmed the presence of positive clones carrying the *AGL42* gene (Supplementary Fig S1). The elimination of the expected 633 bp fragment following restriction digestion with NcoI and BglII enzymes further confirmed the specificity and compactness of the AGL42 plasmid (Supplementary Fig S2). Colony PCR was performed to screen the positive clones of the transformed recombinant vector pCAMBIA_AGL42 in *Agrobacterium* amplifying 506 bp (Supplementary Fig S3). CEMB-100 (*Gossypium hirsutum*) was selected for the *Agrobacterium*-mediated transformation of pCAMBIA_AGL42 based on its germination index. Of the 94 putative transgenic cotton plants, 27 were screened on Murashige and Skoog medium supplemented with hygromycin (with a transformation efficiency of 1.47%), of which only 17 acclimatized transgenic cotton plants survived.

### Molecular and physiological analysis of transgenic cotton plants (T0)

The amplification of 506 bp in eight putative transgenic cotton plants (AG1–AG8) with gene specific primers by PCR confirmed the successful introduction of the transgene in the cotton genome (Supplementary Fig. S4). qRT-PCR of *AGL42* under the 35S promoter showed higher transgene expression in four transgenic cotton plants, namely AG1, AG3, AG4, and AG7. These cotton plants exhibited 23.0-, 113.0-, 32.0-, and 21.6-fold higher expression compared to that of the other transgenic and non-transgenic cotton plants (Supplementary Fig. S5). The detached leaf assay from all transgenic cotton plants determined higher levels of total chlorophyll, chlorophyll a, and chlorophyll b compared to that of the non-transgenic control (Supplementary Fig. S6). However, the transgenic cotton plants AG1, AG4, AG5, and AG7 exhibited 23.11, 29.19, 23.79, and 21.743 mg g-1 FW (fresh weight) total chlorophyll, respectively. This was significantly higher compared to that of the other transgenic and non-transgenic cotton lines (9.11 mg g-1 FW) (Supplementary Fig. S7: A). Similarly, chlorophyll a in transgenic cotton plants AG1, AG4, AG5, and AG7 exhibited 18.66, 22.61, 18.37, and 17.87 mg g-1 FW, respectively. This was significantly higher compared to that of the non-transgenic control (7.51 mg g-1 FW) (Supplementary Fig. S7:B). Chlorophyll b content was recorded as 5.67, 7.84, 5.49, and 4.87 mg g-1 FW, respectively, which was statistically significant than that of the non-transgenic control (1.46 mg g-1 FW) (Supplementary Fig. S7:C).

### Molecular analysis (T1 generation)

Four transgenic cotton plants, AG1, AG4, AG5, and AG7, with the highest transgene expression and chlorophyll content, were raised to the T_1_ generation. Each transgenic and control cotton lines in the T_1_ generation was subjected to PCR analysis, and a product of 683 bp was amplified using a primer set designed from the vector backbone and gene (Supplementary Fig. S8).

### Absolute mRNA quantification

**Figure 1:**
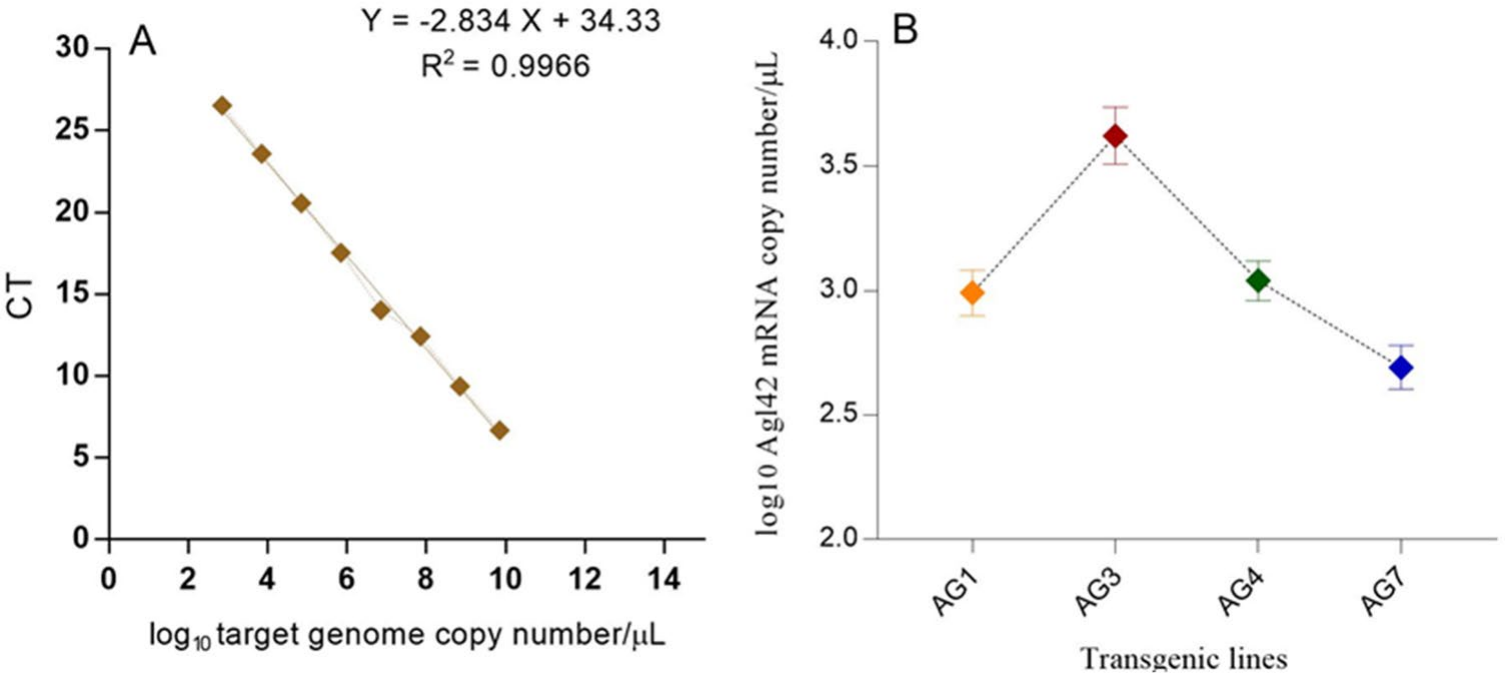
mRNA expression of Agl42 gene in transgenic cotton lines (T1 Generation) (**A**) Standard curve showing log10 values of Agl42 plasmid copy number (**B**) The mRNA copy number of Agl42 gene in different transgenic lines determined by relative absolute quantification.

The absolute mRNA expression of the *AGL42* gene in transgenic cotton lines (T_1_) was determined by plotting a standard curve (Fig. 1a). All values are plotted in log10. Absolute mRNA quantification showed the highest expression of the *AGL42* gene in transgenic cotton line AG3 (3.85) when compared to the log10 values of the standard curve, followed by AG1 (3.17), AG4 (3.01), and AG7 (2.77) (Fig. 1b).

### Determination of GUS reporter gene activity and mRNA expression of 35S_AGL42 in different plant tissues

**Figure 2:**
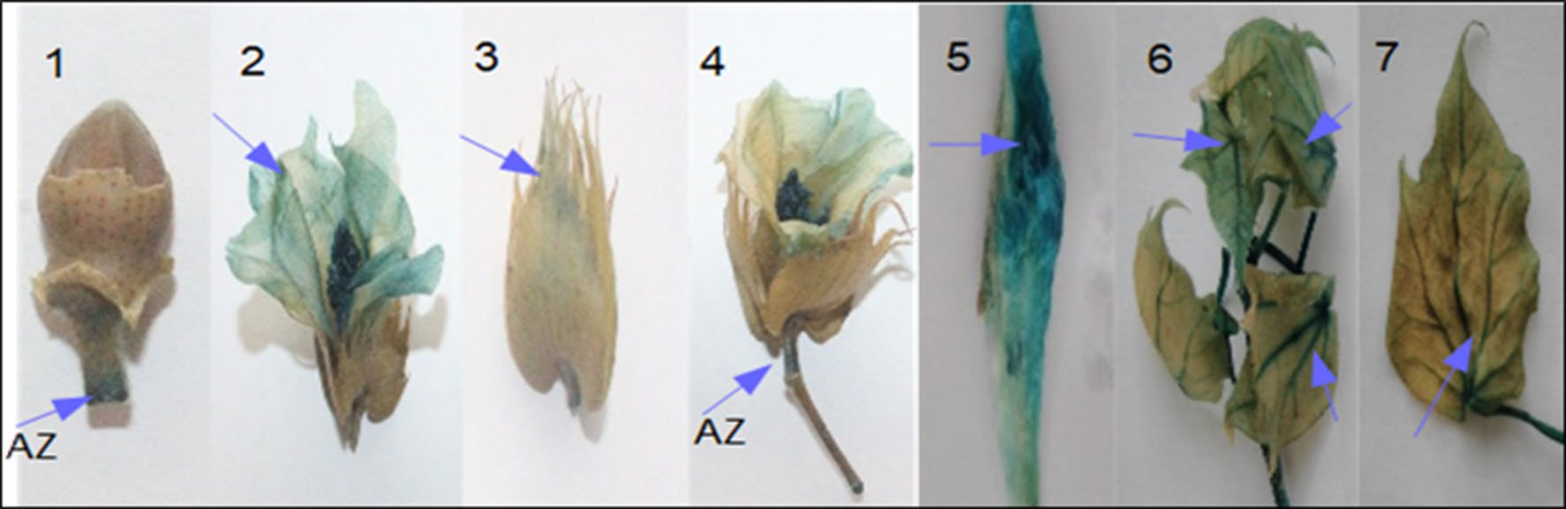
GUS staining of different parts of plants (1) Floral bud AZ (Abscission zone) (2) Petals (3) Sepals (4) flower AZ (5) Fiber (6) Fresh young leaves (7) Senescence leaf.

**Figure 3:**
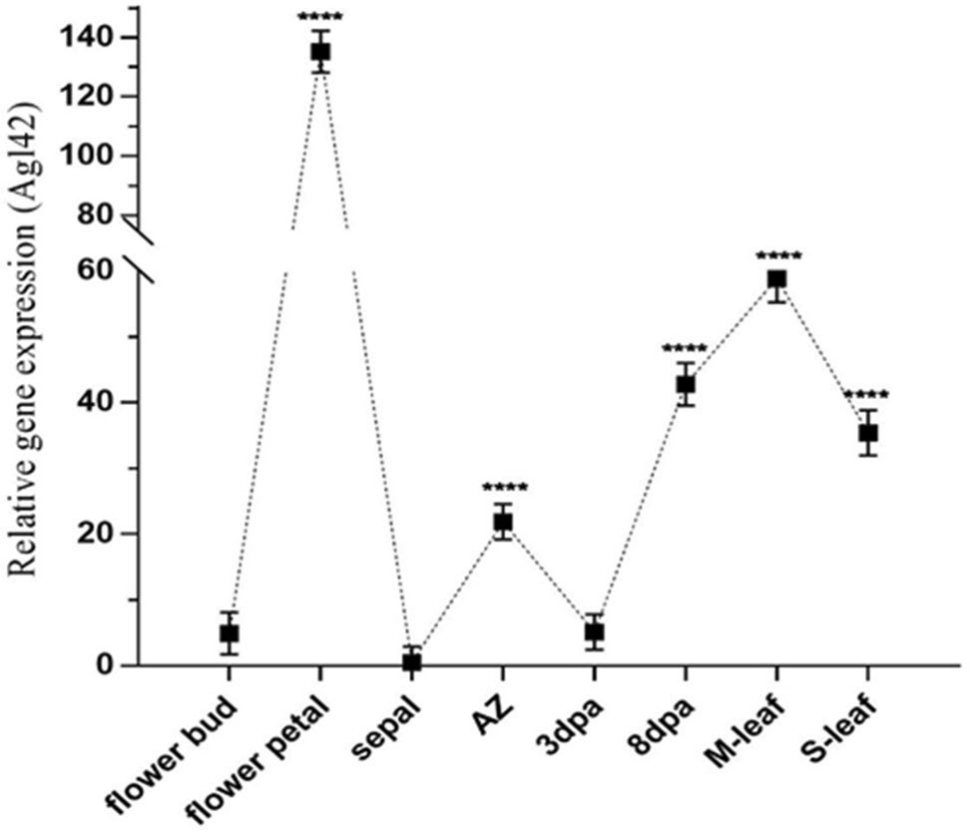
mRNA expression analysis of AGL42 gene in different tissues through qRT-PCR. Each value depicts the average of technical and biological triplicates in AG3 transgenic line. Asterisks specify significant variation “****Pvalue ≤ 0.0001”.

35S_AGL42: GUS activity was evaluated in different tissues of transgenic cotton plants by histochemical assays. The appearance of a greenish blue color in petals, abscission zone (flowers and buds), 8 DPA fibers, fresh mature leaves, and senescent leaves was evident (Fig. 2). Microtomy of leaves and abscission zones of (flower bud) sections were analyzed under microscope at 500 μm, which further confirmed GUS expression activity (Supplementary Fig. S9). To further evaluate the expression of AGL42 under the control of the 35S promoter in different tissues, qRT-PCR was performed using gene-specific primers. The highest expression was estimated in flower petals, followed by fresh mature leaves, 8 DPA fibers, senescent leaves, and abscission zones of flower buds. Differences in the transgene expression in flower buds, sepals, and 3 DPA fibers were not significant (Fig. 3).

### Downregulation of *NAC* senescence genes in drought-stressed *AGL42 *transgenic cotton lines

**Figure 4:**
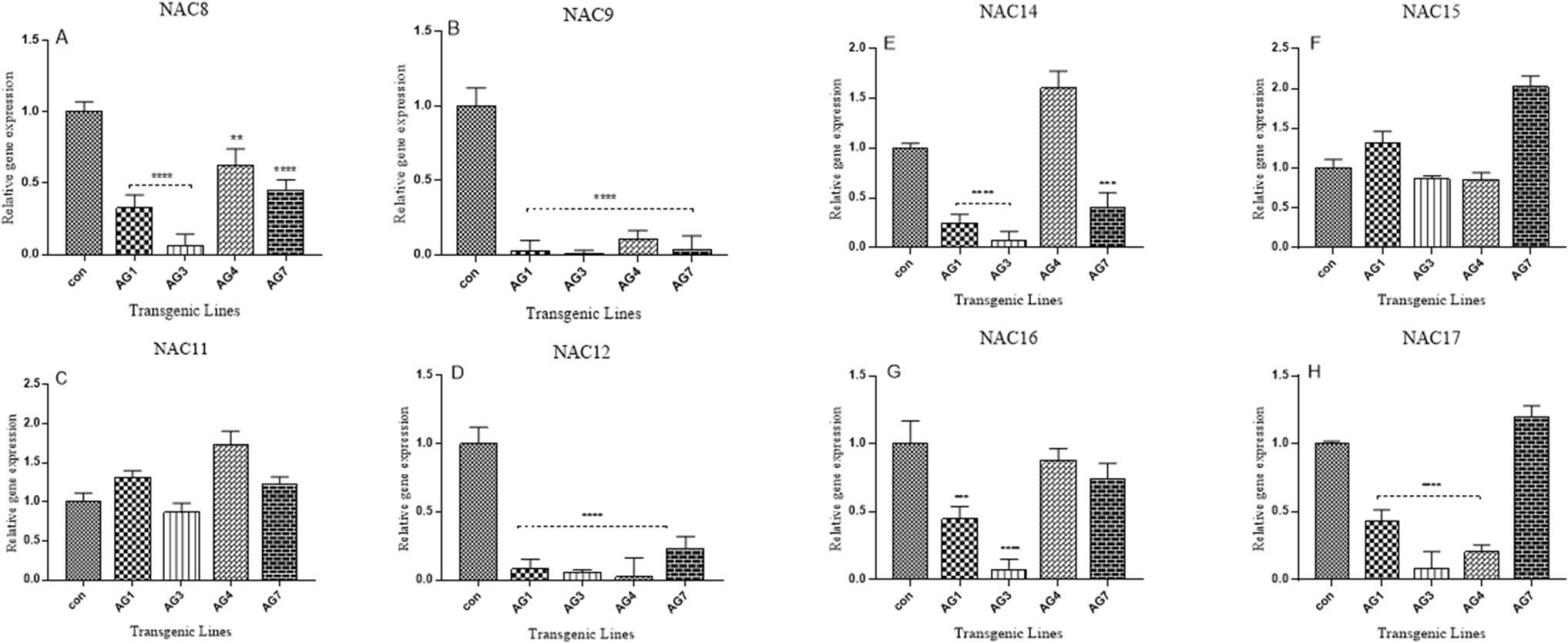
Relative expression of NAC genes (**A**) *GhNAC8* (**B**) *GhNAC9* (**C**) *GhNAC11* (**D**) *Gh NAC12* (**E**) *GhNAC14* (**F**) *GhNAC15* (**G**) *GhNAC16* (**H**) *GhNAC17* in AGL42 transgenic lines All values represent the average of technical and biological triplicate. Asterisks indicate significant difference “****P-value ≤ 0.0001; ***P ≤ 0.001; **P-value ≤ 0.01 through one-way ANOVA”.

**Figure 5:**
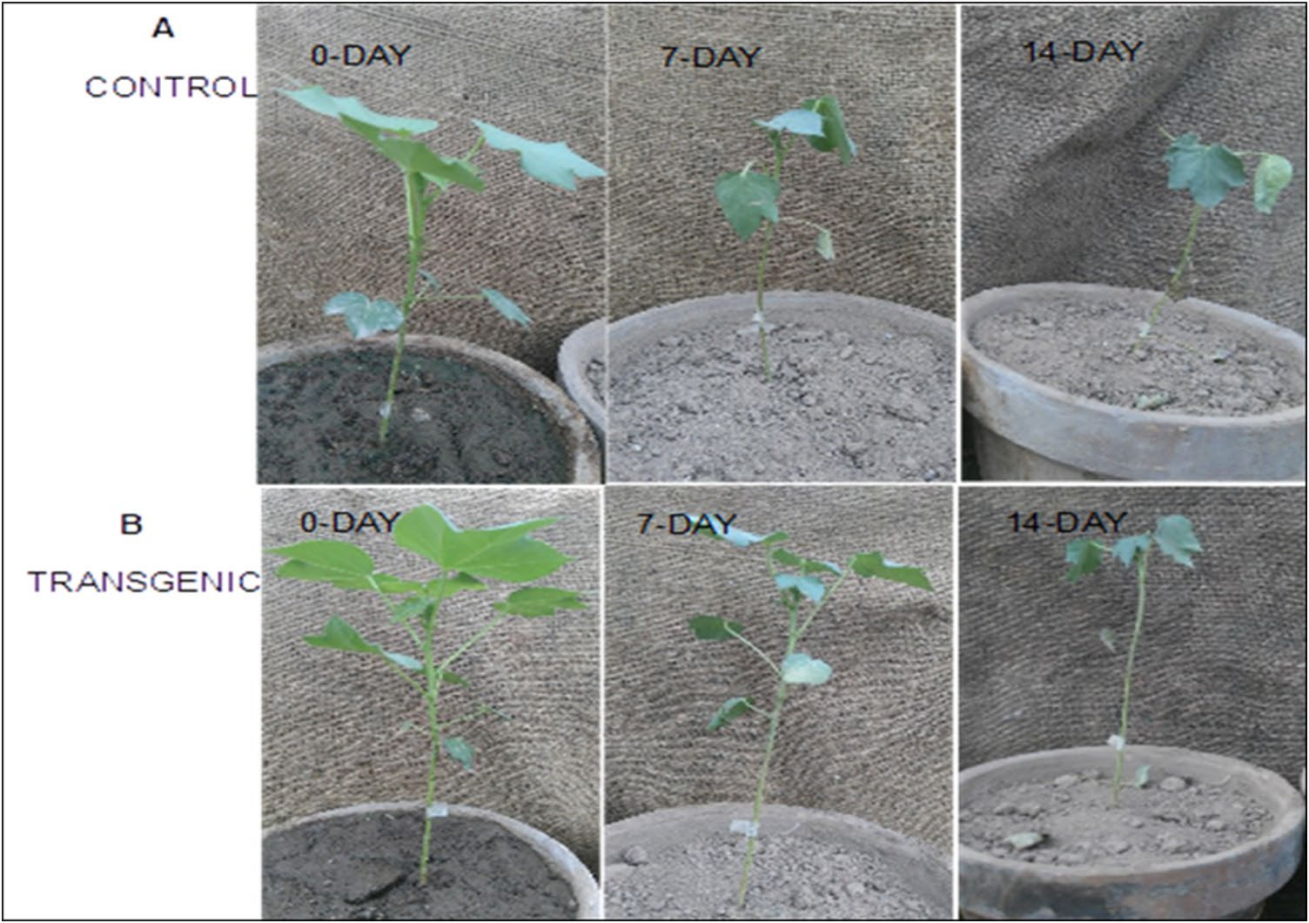
Comparison of drought stress at 0-Day, 7-Day and 14-Day (**A**) Control non-transgenic (**B**) Agl42_transgenic plant.

To determine the effect of *AGL42* as a repressor transcription factor, the relative expression of *GhNAC8*, *GhNAC9*, *GhNAC11*, *GhNAC12*, *GhNAC14*, *GhNAC15*, *GhNAC16*, and *GhNAC17* was measured in a 7-day drought-stressed 35S: AGL42 transgenic and control cotton lines. *GhNAC8*, *GhNAC9*, and *GhNAC12* genes were significantly downregulated in all transgenic cotton lines compared to that of the non-transgenic control (Fig. 4 a, b, d). *GhNAC14* was downregulated in the AG1, AG3, and AG7 transgenic cotton lines (Fig. 4 e); *GhNAC16* was downregulated in the AG1 and AG3 lines (Fig. 4 g); and *GhNAC17* was downregulated in the AG1, AG3, and AG4 lines compared to that of the non-transgenic control line (Fig. 4 h). *GhNAC11* and *GhNAC15* were not downregulated in any of the transgenic cotton lines (Fig. 4c, f.). Both transgenic and non-transgenic plants on day zero of drought stress were similar to the lush green flattened leaves. Wilting symptoms and leaf shedding appeared in non-transgenic control cotton plants on the 7th day with almost complete wilting of leaves occurring on the 14th day of drought stress, whereas transgenic cotton plants remained healthy on the 7th day with comparatively less shedding on the 14th day of stress treatment (Fig. 5).

### In-silico studies of AGL42 protein as a transcriptional repressor

In-silico studies on the downregulation of five *NAC * genes, *NAC8*, *NAC9*, *NAC12*, *NAC14*, and *NAC17*, in AGL42 transgenic plants under drought stress were performed to evaluate the binding affinity of the AGL42 transcription factor with NAC promoters.

### 3D structures of the AGL42 protein and CArG DNA sequences

Functional domains of AGL42 were determined using the Scan Prosite tool, which showed 65 amino acid conserved MADS-box and 185 amino acid conserved K-box domains. The I-TASSER server was used to predict the 3D structure of the AGL42 protein using 1N6J as a PDB template for homology modeling (Supplementary Fig. S10a). The 220 amino acid residues of the AGL42 protein were refined by the MoD refiner. The Ramachandran plot demonstrated that 202 (92.7%) residues of AGL42 were in the favored region, 13 (6.0%) in the allowed region, and three (1.4%) in the outlier region (Supplementary Fig. S10b). Previous reports regarding downregulated *EDF1* and *EDF2* genes in AGL42 transgenic Arabidopsis (Chen et al.^14^) were explored using docking analysis in the current study. Putative CArG boxes (MADS-box consensus binding sequences) were identified in *EDF1* and EDF*2* regulatory region (8,985,150–8,985,159 and 25,943,794–25,943,803) nucleotide sequences, respectively, in Arabidopsis. The location of the CArG sequence in the promoter of *GhNAC* genes was identified (Supplementary Table 1). 3D models of CArG sequences in the NAC promoter region were built using the 3D-DART server (Supplementary Fig. S11A-G).

### Protein-DNA docking of the AGL42 transcription factor against EDFs and NACs

**Figure 6:**
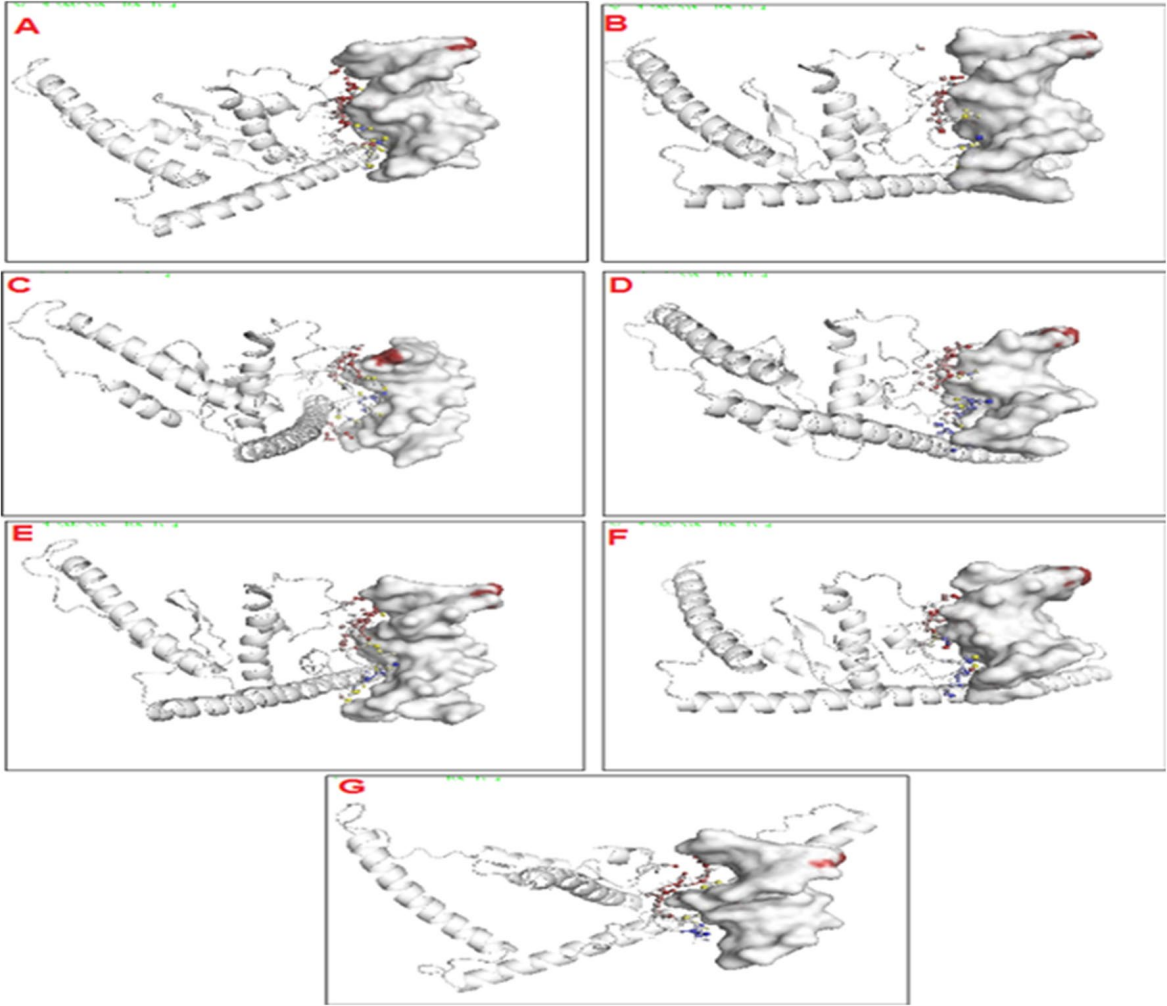
Protein-DNA interface visualizations created by PyMOL plugin PDIviz The sugar-phosphate backbone interface (DNA backbone atoms and protein atoms interacted with them) coloured in red, the DNA bases interface coloured in blue, and atoms/areas involved in simultaneous interaction with DNA bases and backbone coloured in yellow. (**A**) AGl42–EDF1 DNA backbone and bases complex. (**B**) AGL42-EDF2 DNA complex. (**C**) AGl42- NAC8 DNA binding domain complex. (**D**) AGL42—NAC9 DNA complex. (**E**) AGL42—NAC12 DNA binding domain complex. (**F**) AGL42—NAC14 DNA complex. (**G**) AGL42—NAC17 DNA binding domain complex.

The docking analysis showed that amino acid ILE, ASP, LEU, ASN, and LEU residues within the range 199–203 in the IDLNL repressor sequence of the AGL42 protein were involved in the interaction with *EDF1* and EDF2 DNA. During crystal complex formation, ILE and ASP had a greater interaction affinity with the THY residue of EDF1 (Fig. 6a) and ADE residue of EDF2 (Fig. 6b). The evaluation of interface interactions performed by PDBePISA between AGL42 and the NAC8, NAC9, NAC12, NAC14, and NAC17 promoters indicated that ILE, ASP, LEU, ASN, and LEU amino acid residues are involved in the buried surface area of the interface. The results showed that ILE and ASP amino acids from AGL42 had a greater interaction affinity with ADE residues in the NAC8 promoter to form a protein-DNA complex (Fig. 6c). Herein, ILE, ASN, and LEU amino acids of AGL42 had a greater affinity for the THY residue of the NAC9 promoter (Fig. 6d), while ILE, ASP, and LEU from AGL42 had a greater affinity for the THY residue of the NAC12 promoter, forming a packed crystal complex (Fig. 6e). Highly interactive amino acids (ILE, ASP, and ASN) within AGL42 bound to the ADE residue of the NAC14 promoter (Fig. 6f), while ILE, ASP, and LEU had a greater affinity for the THY residue of the NAC17 cotton promoter, forming a protein-DNA complex (Fig. 6g).

Thus, downregulated *EDF1* and *EDF2* genes in AGL42 transgenic Arabidopsis exhibited strong interactions with ILE199 and ASP200 amino acids within the repressor sequence of AGL42 protein in docking analysis. These results were consistent with the AGL42 protein docked with cotton NAC promoters where ILE199 and ASP200 amino acid residues of the AGL42 protein exhibited the highest interaction affinity to the CArG sequences in the promoters of cotton *NAC* genes. These docking results support the binding and suppression of NAC genes in transgenic cotton lines, which has already been shown in mRNA expression results. Hence, transcription factor AGL42 acts as a broad-spectrum repressive protein.

### Photosynthesis and chlorophyll content in drought-and ABA-stressed transgenic cotton plants (T1)

**Figure 7:**
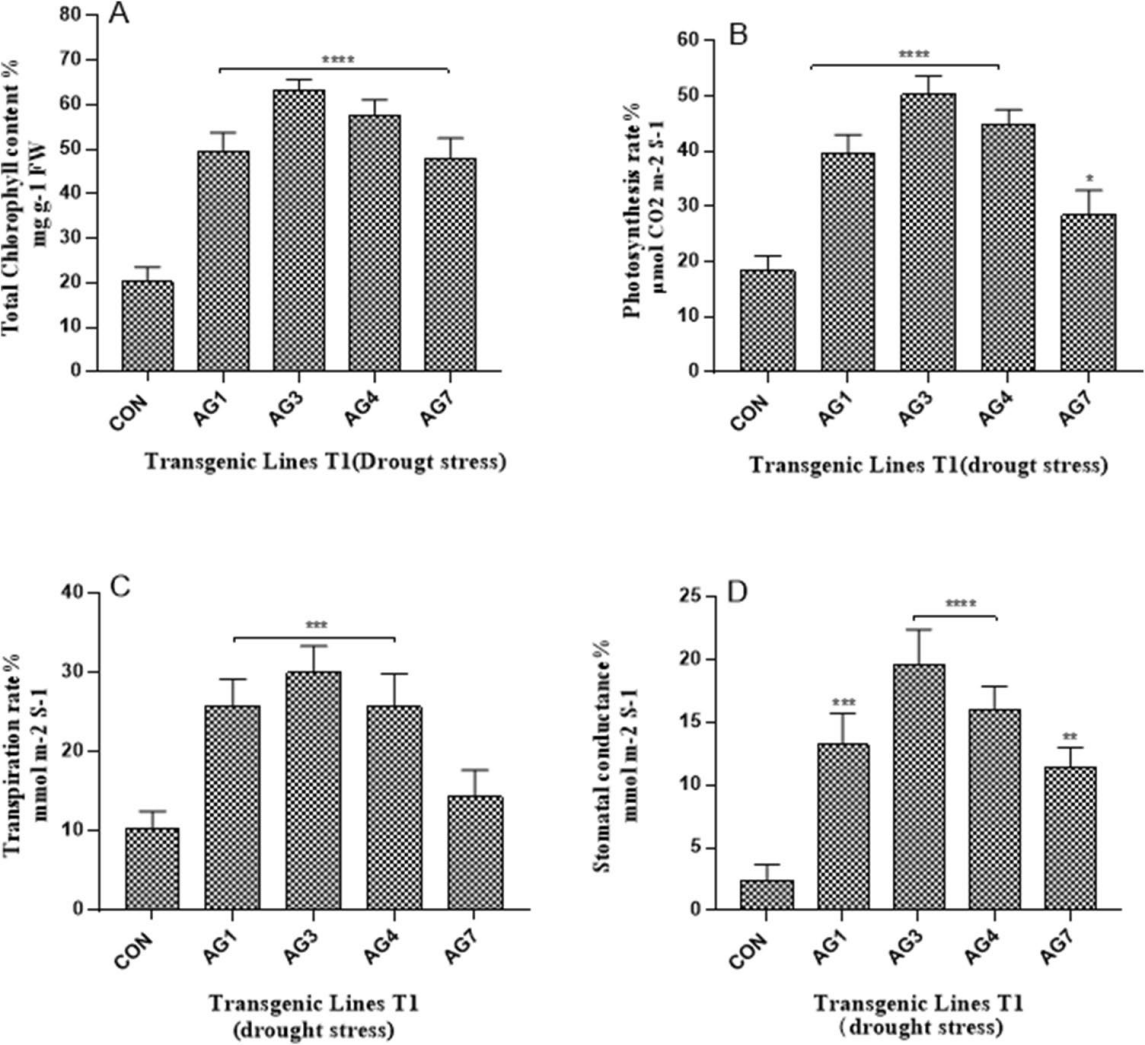
Percentage estimation before and after drought stress in leaves of control and transgenic lines. (**A**) Total chlorophyll contents %; (**B**) Photosynthetic rate %; (**C**) Transpiration rate %; (**D**) Stomatal conductance %. Each bar depicts the average of biological triplicates from control and transgenic lines. Asterisks specify significant variation: “****P value ≤ 0.0001; ***P ≤ 0.001; **P value ≤ 0.01 *P value ≤ 0.05”.

**Figure 8:**
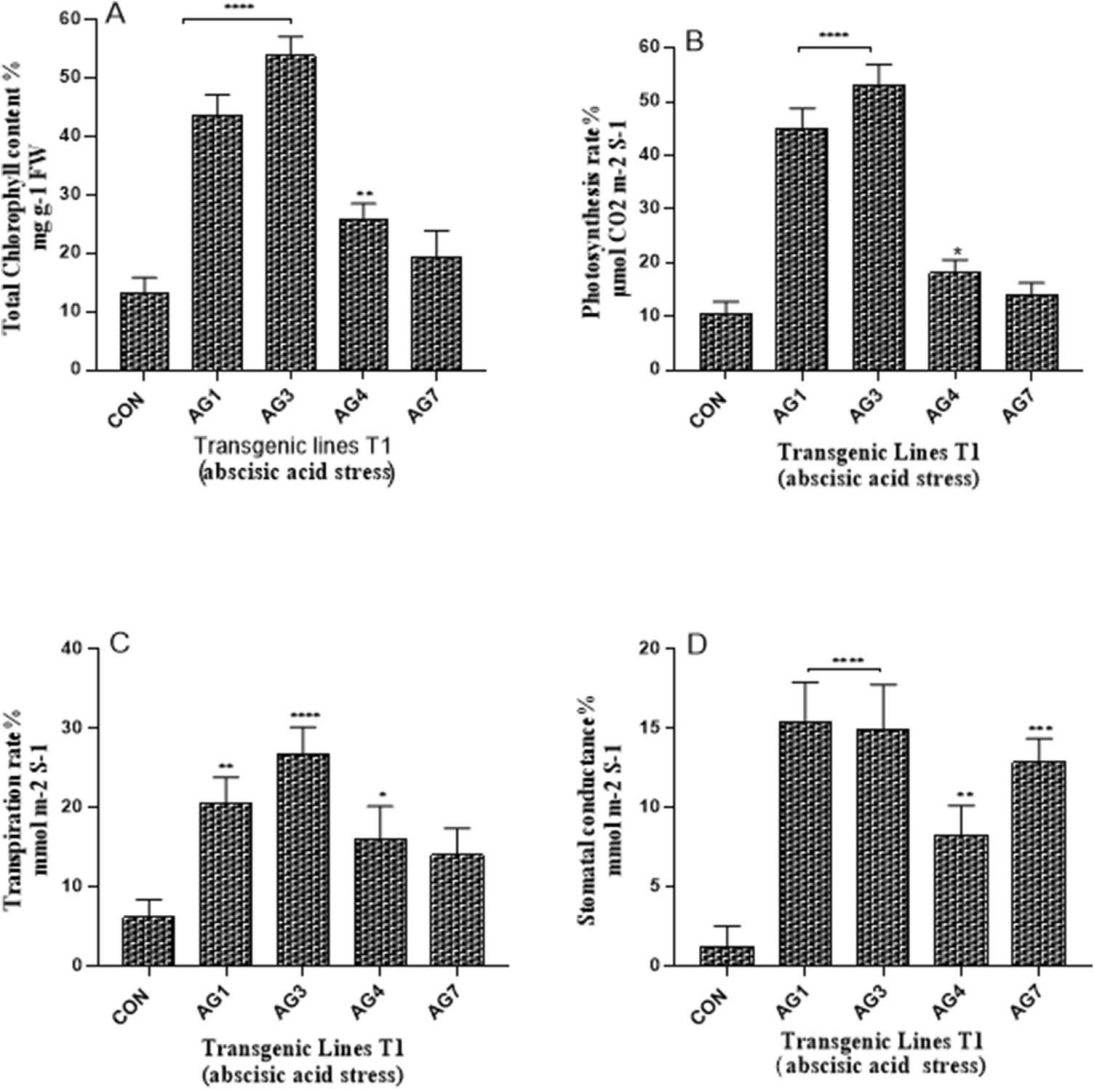
Percentage estimation before and after abscisic acid stress in leaves of control and transgenic lines. (**A**) Total chlorophyll contents %; (**B**) Photosynthetic rate %; (**C**) Transpiration rate %; (**D**) Stomatal conductance %. Each bar depicts the average of biological triplicates from control and transgenic lines. Asterisks specify significant variation: “****P value ≤ 0.0001; ***P ≤ 0.001; **P value ≤ 0.01 *P value ≤ 0.05”.

The total chlorophyll, photosynthetic rate, transpiration rate, and stomatal conductance in transgenic cotton lines AG1, AG3, and AG4 calculated after 14 days of drought stress were significantly higher than those in the control cotton line. Among these transgenic cotton lines, AG3 showed the highest percentage of chlorophyll (63%), photosynthetic rate (50%), transpiration rate (30%), and stomatal conductance (19%) compared to those in the control cotton line, which exhibited 20%, 18%, 10%, and 2%, respectively, after 14 days of drought stress (Fig. 7). In contrast, 14-day ABA treated transgenic cotton lines AG1 and AG3 exhibited significantly higher total chlorophyll content and photosynthetic rate than that of the non-transgenic control. AG3 showed the highest total chlorophyll content (54%), photosynthetic rate (33%), transpiration rate (27%), and stomatal conductance (15%) compared to those of the non-transgenic control, which exhibited 13%, 10%, 6%, and 1.21%, respectively, after ABA stress treatment (Fig. 8). These results are in accordance with the relative mRNA expression of *AGL42*, which was the highest in the AG3 transgenic cotton line.

### Morphological and fiber traits of the AGL42 transgenic cotton plants (T_1_)

**Figure 9:**
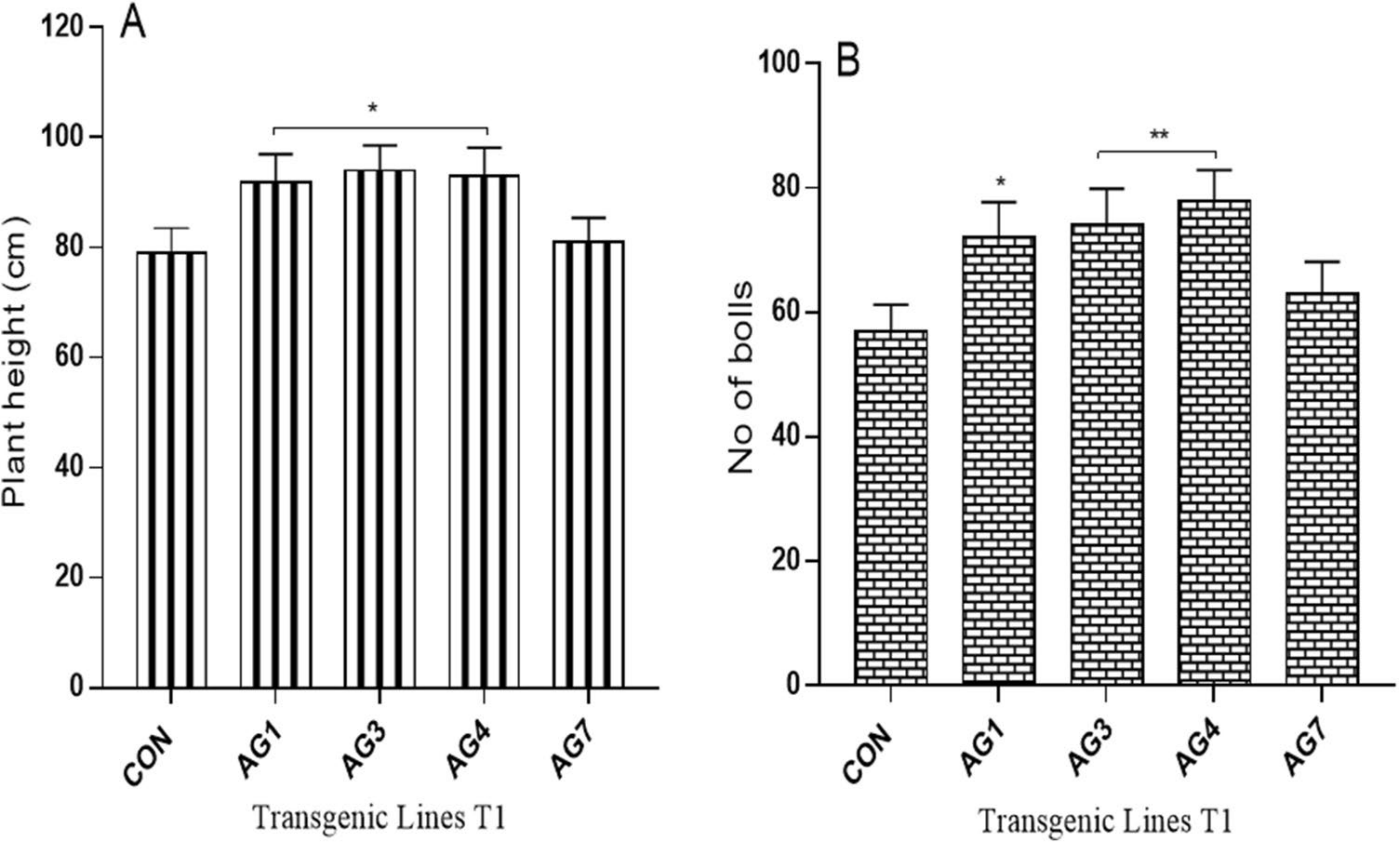
Morphological traits of transgenic and control plant lines (T1) (**A**) Plant height: (**B**) No of bolls: each bar signifies the average of biological triplicate from each control and transgenic line.

The average heights of transgenic cotton lines AG1, AG3, and AG4 were 92, 95, and 93 cm, respectively, which were significantly higher than those of the non-transgenic control at 79 cm (Fig. 9a). The average number of bolls in transgenic cotton lines AG1, AG3, and AG4 was 72, 74, and 78, respectively, which was significantly higher than those of the non-transgenic control (57) (Fig. 9b). These results showed higher retention of floral buds, squares, and young bolls in the transgenic cotton lines than those in the non-transgenic control. However, the fiber traits of all transgenic cotton lines were statistically non-significant compared to that of the non-transgenic control. There was a negligible difference in the fiber length (25.2 mm), strength (28.6 (g/tex)), and micronaire value (3.67) of the AG3 transgenic cotton line compared to those of the control (23.1 mm, 26.9 (g/tex), and 4.1, respectively) (Supplementary Fig. S12).

### Determination of chromosomal location of the *AGL42* gene in T2 generation

**Figure 10:**
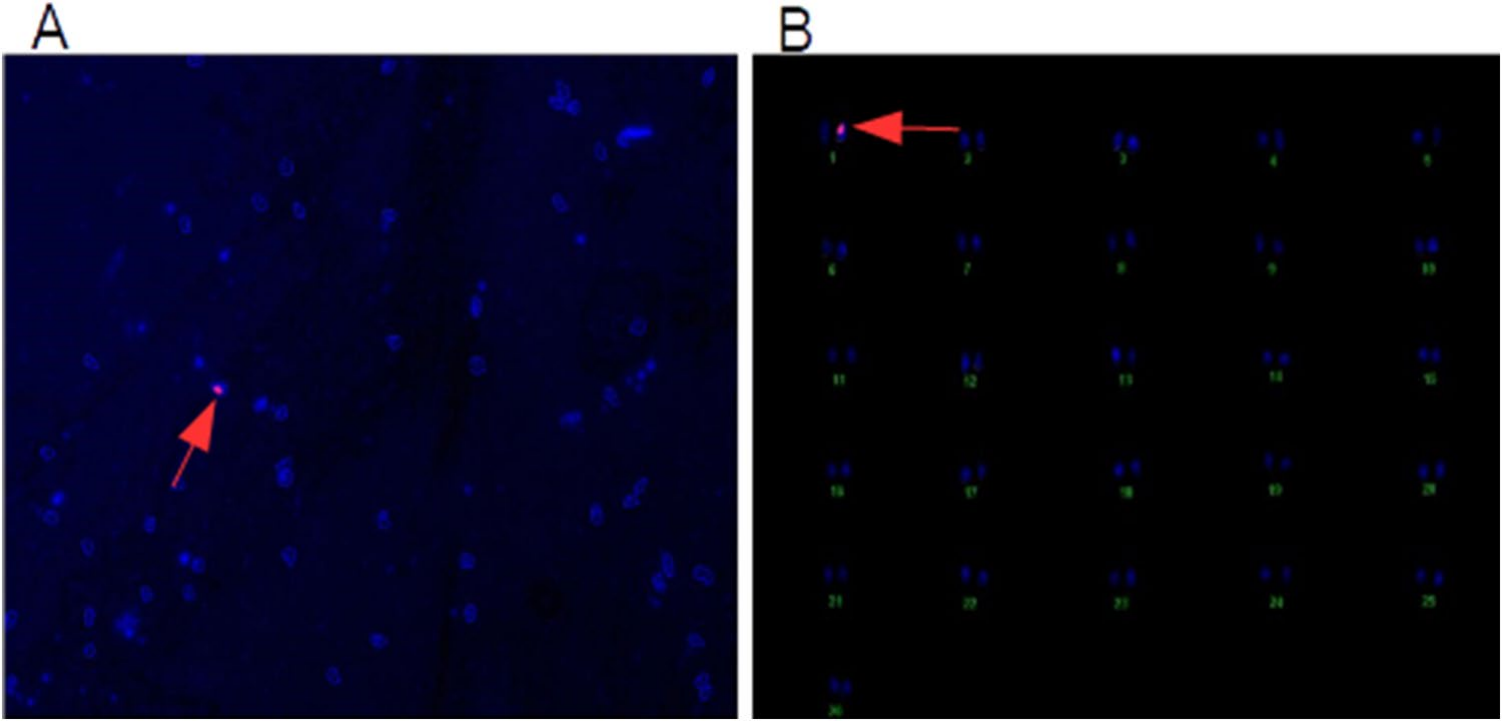
(**A**) Agl42 probe hybridization; (**B**) Karyotyping and AGL42 location on chromosome 1 in cotton genome specified by fluorescent signal.

The AG3 transgenic cotton line with the highest transgene expression and delayed premature leaf senescence was subjected to transgene localization using fluorescence in situ hybridization (FISH) in the advanced T2 generation. The appearance of a fluorescent signal on chromosome 1 in the hemizygous form, indicated by the arrow, depicts the successful integration of the AGL42 gene in the cotton genome (Fig. 10).

## Discussion

Overexpression of the *AGL42* gene in cotton delayed leaf senescence by downregulating senescence-causing *NAC* genes. Premature leaf senescence is one of the major factors restraining cotton production^20^. Herein, the transcription factor AGL42 (FYF), a repressor protein cloned into the plant expression vector pCAMBIA1301 under the 35S promoter with Nco1 and BglII restriction enzymes, was used to delay leaf senescence in cotton because of its ability to delay floral senescence in Arabidopsis^14,16^ and fruit, sepal, and leaf senescence in tomato^18^. The pCAMBIA 1301 high-copy noplant expression vector for cotton transformation was used as previously described^21–24^. The transformation efficiency in *G. hirsutum* was 1.47%, which was almost similar to 1.32%^24^, and this may be attributed to similar methodology and crop response^24^.

Eight transgenic cotton plants AG1–AG8 carrying the *AGL42* gene in the T0 generation were confirmed through amplification of 506 bp using gene-specific primers. The relative expression of *AGL42* under the constitutive promoter in transgenic cotton plants was higher, particularly in AG1, AG3, AG4, and AG7, when compared with that of the non-transgenic control, which might depend on the site of insertion and copy number as reported previously^25–27^. The CaMV35S promoter also plays a vital role in the variable expression of transgenes in different plant transformants^28,29^.

An assay for the leaf detachment of stressed cotton plants was conducted for 7 days to observe the chlorophyll content of transgenic and non-transgenic cotton plants. The total chlorophyll, chlorophyll a, and chlorophyll b contents were comparatively higher in the AG1, AG3, AG4, and AG7 plants than those in the other transgenic and non-transgenic cotton lines. These results coincide with the findings of Chen et al., (2011)^14^, which showed sustained green color in AGL42 transgenic Arabidopsis leaves after 5 days of the leaf detachment test. Similar results were also observed in Arabidopsis overexpressing CmBBX22^30^, which resulted in delayed leaf senescence and improved drought tolerance. The transgenic cotton plants showed higher chlorophyll content with elevated relative expression of *AGL42* in AG1, AG3, AG4, and AG7, which was raised to the T1 generation. qPCR analysis for absolute quantification was also performed to determine the exact copy number of AGL42 mRNA in different transgenic cotton lines, and different copy numbers were obtained ranging from the log10 gene copy number of 2.7 in the AG7 cotton line to 3.8 in the AG3 line. Consistent with earlier studies, the use of qPCR to calculate gene copy number in different transgenic plant lines provides accurate data^31–33^.

In the 35S_AGL42: GUS transgenic cotton line, GUS activity was observed in different tissues showing strong expression in petals, young and mature leaves, the abscission zone of young flower buds, and mature flowers, whereas very little expression was observed in sepals. The microtomy results of the leaf and abscission zones of (flower bud) sections further verified that the GUS expression level decreased in the same range as mentioned above. Similar results were obtained by Chen et al.,﻿ (2011) who determined the expression of the GUS: FYF (AGL42) gene under the 35S promoter in different flowering parts of Arabidopsis^14^. The results were also consistent when evaluated on a relative basis through mRNA expression of AGL42 in different tissues, which showed the highest expression in petals, followed by leaves, immature fiber (8 DPA), and AZ of floral parts, as described earlier by Xie et al.^18^. The results demonstrated that the expression of AGL42 as a repressor protein in different tissues was effective in delaying premature senescence, as reported previously^16,18^.

Ten *GhNAC* genes (*GhNAC8*–*GhNAC17*) were differentially expressed during natural and induced leaf senescence. qRT-PCR analysis revealed the upregulation of these *GhNAC* genes in senesced leaves under ABA, ethylene, drought, salinity, cold, heat, and other hormonal stresses^6^. To determine the mechanism of delaying premature leaf senescence under certain stresses, the impact of *AGL42* expression on *NAC* genes was investigated. The presence of “IDLNL” repressor sequence in the AGL42 protein has already been demonstrated in the downregulation of *EDF1* and *EDF2*. *BOP1* and *BOP2* genes are involved in promoting senescence in floral organs^14^. We performed in silico docking of the IDLNL repressor sequence of the AGL42 protein with the promoter of *NAC* genes. CArG box is the cis-regulatory element of the MADS domain of AGL42 in target promoters^34^. Interestingly, CArG-box has also been identified in the promoter regions of *GhNAC8*, *GhNAC9*, *GhNAC12*, *GhNAC14*, and *GhNAC17* leaf senescence promoting genes. Hence, In order to compare their binding affinity, the 3D structure of the AGL42 protein was also docked with CArG binding sequences in the promoter regions of *EDF1* and *EDF2*, which already has been reported to be repressed by the AGL42 protein^14^. In all docking results, ILE199 and ASP200 residues fell within the range of 199–203. The (IDLNL) repressor sequence in the AGL42 protein showed strong interaction with the promoter regions of *EDF1* and *EDF2* in Arabidopsis, which was consistent with AGL42 protein docked with cotton NAC promoters. This suggests that *NAC* genes might be downregulated by the AGL42 repressor protein. Our docking results were consistent with those of previous studies^35–37^.

A 7-day drought stress assay to study the relative expression of NAC genes in T1 transgenic and non-transgenic cotton lines was performed. The results demonstrated that *GhNAC8*, *GhNAC9*, *GhNAC12*, *GhNAC14*, *GhNAC16*, and *GhNAC17* were downregulated in AG1 and AG3 transgenic cotton lines compared to that of the non-transgenic control; however, variable results regarding the expression of the above-mentioned *NAC* genes were observed in AG4 and AG7 transgenic cotton lines. These results are consistent with those of previous findings^14,18^, in which downregulation of senescence-promoting genes was evident in over-expressed *FYF *(*AGL42*) in Arabidopsis and tomato plants^18^.

Shah et al. (2013) and Jan et al. (2019) reported that the overexpression of *NAC * genes under drought and ABA-stress is responsible for the degradation of chlorophyll content, which promotes early leaf senescence^6,38^. In the present study, the percentage of total chlorophyll content in transgenic cotton plant leaves with downregulated *NAC* genes after 14 days of drought stress was estimated to be 51%, 63%, and 49% in AG1, AG3, and AG4, respectively, compared to those in the control leaves, which had a 20% higher photosynthetic rate. These results are in accordance with those of Fan et al., (2015) who reported that silencing of the *GhNAP* gene in cotton resulted in higher chlorophyll content and net photosynthetic rate compared to that of the wild type^8^.

ABA-induced NAC TFs are involved in the earlier senescence of leaves^39^; therefore, in the current study, the leaves of 35S_AGL42 transgenic cotton lines with downregulated *NAC* senescence genes had higher total chlorophyll content, which was measured before and after 14 days of ABA stress. Within the transgenic cotton lines, the total chlorophyll content in AG1 and AG3 was found to range between 43 and 53%, respectively, compared to that of the control cotton line (19%). These results are in accordance with the study by Guo and Gan^40^, which showed that NAC TFs promote leaf senescence in Arabidopsis, and as reported by Liang et al., downregulation of NAC genes in rice results in delayed leaf senescence^41^.

Agronomic and fiber quality parameters were also measured to evaluate the impact of the transformed negative regulator. Among the transgenic cotton plants vs. non-transgenic controls, we found no significant difference in fiber length, strength, micronaire value, and uniformity index, except for increased plant height and number of bolls, owing to no direct correlation, as was evident from the literature. However, Fan et al. (2015) reported that while cotton lines with downregulated *GhNAP* gene showed no effect on plant height, it reduced fiber length up to 7%, contradicting the findings of this study, which might be because of the genotype response of the plant used in their experiments^8^. The higher number of bolls in AGL42 transgenic cotton plants is owing to its role in floral bud and square (young bolls) retention, which is in accordance with an earlier report by Chen et al. (2011)^14^ in Arabidopsis and Xie et al. (2014) in tomato plants^18^. The number of bolls is a primary determinant of cotton yield, hence, an increase in number of bolls would represent improvement in cotton fiber yield in AGL42 transgenic cotton plants^42^. These results are in line with the study by Fan et al. (2015), which reported a percent lint yield improvement of 41.79% in GhNAPi cotton lines compared to that of the wild type (38.08%)^8^. FISH of this line in the T2 generation determined the location of the *AGL42* gene on chromosome 1, as determined by Liu et al. (2020)^43^.

## Conclusion

The AGL42 transgenic cotton lines with downregulated *NAC* senescence genes were found to have higher total chlorophyll contents in stressed condition. The higher no of bolls in AGL42 transgenic cotton plants is owing to its role in floral buds and squares (young bolls) retention. The results are supportive to use these promising cotton lines in breeding program of variety development for higher yield.

## Materials and methods

### Chemical synthesis of codon optimized anti-senescence gene *AGL42*

The sequence of *Arabidopsis* mad box transcription factor *AGL42* (*FYF*) was retrieved from Gene Bank (Accession number AY141213.1) and preferred codons were optimized according to the genome of cotton (*Gossypium hirsutum*) using online Integrated DNA Technologies (IDT) tool. The integrity of these codon optimized CDS sequences was evaluated to confirm the suitability of expression cassette frame (Start and stop signals) using available bioinformatics software for protein analysis i. e. ExPASy. The chemical synthesis of these sequences required appropriate selection of non-cutter enzymes which were analyzed by an online available WEB-CUTTER 2.0 tool. The codon optimized *At* *AGL42* gene was synthesized commercially within pUC57 and its NcoI and BglII restriction enzymes were used for its characterization. The online available software SnapGene (viewer 4.3.4) was used to confirm the proper structure and function of protein domains of AGL42 fused with GUS reporter gene.

### Preparation of recombinant plasmid

The codon optimized *AtAGL42* gene was later ligated in plant expression vector pCAMBIA-1301 at NcoI and BglII restriction sites. The Recombinant binary vector which comprised of AtAGL42 gene fused with GUS under 35S constitutive promoter, was transformed into top-10 (*E. coli*) competent cells having the selection of tetracycline (12.5 mg/mL) and kanamycin (50 µg/mL). The confirmation of true ligation for *AtAGL42* was done through PCR amplification and restriction digestion by using gene specific primers fast digest Nco1 and BglII enzymes. The positive clone of recombinant vector pCAMBIA1301_ AtAGL42, was transformed into freshly prepared LBA4404 competent cells through electroporation procedure using Bio-Rad Gene Pulser (Model:165–2105). The bacterial colonies of *AGL42* gene construct were screened through colony PCR by using gene specific primers.

### *Agrobacterium* mediated transformation of cotton through shoot apex cut method

Different varieties of *Gossypium hirsutum* CEMB-66, CEMB-100, CEMB-88 and CEMB-33 were acquired from the CEMB repository, University of the Punjab and subjected to determine the germination index to choose the best germinated variety for transformation of pCAMBIA_AtAGL42. The mature embryos were isolated from de-linted cotton seeds (CEMB-100) and were subjected to *Agrobacterium* mediated transformation using shoot-tip-cut method^44^. The treated embryos were incubated in overnight grown *Agrobacterium* culture having recombinant plasmid (OD600 = 1.0) and were harvested at 3000 rpm before resuspension in MS-broth. After 2 h of incubation at 28 °C the explants were dried and co-cultivated on MS-0 medium plates containing cefotaxime (100 mg/ml). The healthy plantlets were shifted to MS tubes supplemented with hygromycin (25 µg/ml), B5-vitamins and cefotaxime (100 mg/ml) (28 °C; 16 h photoperiod) after three days. After two-weeks of hygromycin screening, the survived cotton plants were transferred into soil pots covered with polythene bags and underwent the process of acclimatization until it can withstand more than six hours of bright sunlight. The plants were finally shifted to soil in containment for further evaluation.

### PCR confirmation of transgenic cotton plants

The genomic DNA of putative transgenic cotton plants (T0) was isolated by CTAB method followed by PCR amplification which was processed using gene-vector detection primers. The flowers of positive transgenic cotton plants were covered with butter paper to proceed with selfing which was done for homozygous lines development. The advanced lines of T1 and T2 generation were also screened for pCAMBIA_AtAGL42 detection through PCR.

### Quantitative real time PCR of G. hirsutum_AGL42 cotton plants (T0)

Purified RNA was extracted from leaf samples of 2.5 months old transgenic and non-transgenic cotton plants (T0 generation). DNA-free Kit (cat # AM1906) was used to decontaminate the freshly isolated RNA samples. The CDNA was synthesized by using Thermo Scientific “First Strand cDNA Synthesis Kit” (Cat # K1622) with hexamer primer. Quantitative Real Time PCR was performed to evaluate the expression of *AGL42 gene*. Total 20 μL reaction mixture was prepared by adding 200 ng of cDNA template, 0.2 μM of each primer Agl42-F(RT) and Agl42-R(RT), and10 μL Thermo Scientific: Maxima SYBR Green/ROX (Cat# K0221). The Actin gene (*GhAct-4*) was used as an internal control. Each sample was proceeded in triplicate. Delta CT method was used to calculate the relative expression levels which were then statistically analyzed and graphically represented by GraphPad Prism 7^45^. One analysis of variance (ANOVA) was applied to determine the significant differences between the control and transgenic cotton lines. Significant differences were considered at p > 0.05. All primer sequences for PCR gene detection, real time and GhAct-4 were listed in supplementary table2.

### Absolute mRNA quantification of G. hirsutum_AGL42 cotton plants (T1)

The seeds of plants having highest transgene expression were collected and the T1 generation was cultivated and evaluated for absolute expression of AtAGL42 gene. The known concentration of the plasmid DNA was diluted into serial dilution up to seven-fold (7.043 × 10^9^ to 7.043 × 10^2^). Total 1µL of each dilution was used in triplicate for quantitative real time (qPCR) using gene specific primers to obtain the Ct (threshold cycle) values. The standard curve was plotted by using those values. Simultaneously, 1µL cDNA from the transgenic cotton plants was also evaluated to calculate the exact mRNA copy number by interpretation of the threshold cycle (Ct) values with respect to the corresponding standard curve using the formula; Ct = Kx + b, where K is the slope value, b is the intercept value and x is the unknown value of log_10_ target genome copies. The result was expressed as log_10_ target genome copy number/ µL of plasmid DNA.

### Gus assay and relative expression of 35S_AGL42 in different tissues (T1)

The transgenic cotton line showing highest transgene expression and best physiological features was evaluated for expression pattern of AGL42 by using GUS staining of different tissues with β-glucuronidase. The plant material was incubated for 12–36 h at 37° C for GUS analysis. Microtomy was performed to further analyze the GUS stain in leaf and stem parts. The tissues were submerged with 10% formalin fixative solution at 4 °C overnight. The samples were subjected to 2 h successive dehydration in 70%, 80%, 90% and 100% ethanol at 4 °C. The leaf and stem tissues were treated with xylene for 30 min and embedded in molten paraffin in metal block which was kept at 4 °C overnight. About 7–15 µm thick tissue sections were cut by Microtome: HM 340E Electronic Rotary (Thermo Scientific, USA) and mounted onto gelatin-coated slides which were analyzed under microscope (Olympus BX61, USA) at 200 μm and 500 μm.

The GUS assay was co-related with mRNA transcript of *AGL42* gene in flower bud, flower petal, sepal, abscission zone, 3DPA, 5DPA, mature leave and senesced leave through q-RT PCR described above using GAPDH as housekeeping control.

### Experimental stress intervention and expression of GhNAC genes in 35S:AGL42 transgenic cotton plants

A group of three plants (2 months old) within each transgenic cotton line and non-transgenic control cotton lines were subjected to water stress while three plants from same lines were exposed to abscisic acid stress up to 14 days. Abscisic acid (Sigma-Aldrich # A4906) used in experiment was dissolved in ethanol (10 mg/mL). The clear solution of abscisic acid was further diluted up to 200 μM and sprayed on leaves of both transgenic and control cotton plants. All stress treatments were carried out under controlled conditions of green house at CEMB. The relative mRNA expression of the senescence regulating *NAC* genes *GhNAC8* (JQ914139), *GhNAC9* (JQ914140), *GhNAC11* (JQ914142), *GhNAC12* (JQ914143), *GhNAC14* (JQ914145), *GhNAC15* (JQ914146), * GhNAC16* (JQ914147), *GhNAC17* (JQ914148) was measured in all 35S_ AGL42 transgenic cotton plants along with non-transgenic control after 7 days of drought stress treatment. *G. hirsutum* Actin gene was used as an internal control to normalize the data. The RT-primer sequences for *NAC* genes were listed in supplementary table 2.

### Molecular docking analysis of AGL42 transcription factor with ethylene response DNA-binding factors (EDFs) of Arabidopsis and NACs promoters of cotton plant

The “IDLNL” repressor sequence in AGL42 transcriptional factor, has its role in down-regulation and decrease in expression of EDF1and EDF2 genes by suppression of the ethylene response. To determine the bioinformatic mechanism, molecular docking was done to illustrate the most interacting amino acid residues in IDLNL sequence that bind to CArG sequence in promoter of *EDF1* and *EDF2* genes from Arabidopsis and compared with *GhNAC8*, *GhNAC9*, *GhNAC12*, *GhNAC14*, and *GhNAC17* genes of cotton to further validate expression results.

The codon optimized *AGL42* gene was translated into amino acid sequence by using expasy translate tool (https://web.expasy.org/translate/). Three-dimensional modelling of AGL42 based on homology modelling and threading was used by online I-TASSER server (https://zhanglab.ccmb.med.umich.edu/I-TASSER/). The PDB template used 1N6J for homology modelling of AGL42. The model was further refined by using Mod Refiner online tool from the Zhang Lab website (https://zhanglab.ccmb.med.umich.edu/ModRefiner/). The protein model was evaluated and validated by a Ramachandran plot. The Ramachandran plot was created by online tool RAMCHANDRAN plot analysis (http://mordred.bioc.cam.ac.uk/~rapper/rampage.php). Various physical and chemical parameters of 3D model of AGL42 transcription factor were determined by using Prot Param online tool (https://web.expasy.org/protparam/). Promoter sequences of *EDF1* (Accession # At1g25560) and* EDF2* (accession # At1g68840) genes were retrieved from Arabidopsis *cis*-regulatory element database (http://agris-knowledgebase.org/AtcisDB/) and NAC promoters sequences, NAC 8 (accession # JQ969023), NAC 9 (accession # JQ969024), NAC 10 (accession # JQ969025), NAC 14 (accession # JQ969029), and NAC 17 (accession # JQ969032) were also retrieved from NCBI and their Putative CArG boxes (MADS-box consensus binding sequences) were identified. The 3D structures of DNA duplex of EDFsand NACs were predicted through 3D-DART server (http://milou.science.uu.nl/services/3DDART/). Protein-DNA docking of all promoter DNA models with AGL42 protein was carried out by using HADDOCK2.2 (http://milou.science.uu.nl/services/HADDOCK2.2/haddockserver-easy.html) online bioinformatics server. HADDOCK2.2 server is best known for protein-DNA docking. To analyze the interaction between the protein and DNA duplexes, PDBePISA (http://www.ebi.ac.uk/msd-srv/prot_int/cgi-bin/server-bin/server) was used. Furthermore, Pymol software was used to visualize the protein-DNA complexes.

### Leaf detachment test for senescence

Three mature leaves from each 35S:AGL42 transgenic and non-transgenic control cotton plants (T0 generation, 2 months old) were carefully excised and kept on three wet layers of filter paper which were placed molded in petri plates (16 cm). Those plates were covered with aluminum foil and were placed in the dark for 7 days under 25° C air condition (AC) for continuous flow of air. Total chlorophyll, chlorophyll a and b pigments were also assessed by protocol described by Xie et al*.*, 2014^18^ by grinding 7 day detached leaves in liquid nitrogen and immersion in 10 ml solution of 80% acetone: ethanol (3:1) and incubated in dark for 48 h. The chlorophyll contents were measured in triplicate from each extracted plant sample. The extracted suspension was centrifuged (4 °C) at 3000 rpm for 10 min and the absorbance was measured by Bio-Rad Spectrophotometer; xMark™ at 645 nm and 663 nm. The formula based on the method of Arnon, 1949^46^ was used to calculate the chlorophyll contents.

### Determination of photosynthetic rate and chlorophyll contents of drought and abscisic acid stressed transgenic cotton plants

The photosynthetic rate along with transpiration rate and stomatal conductance of each cotton plant (T1 generation) before and after 14 days interval of drought and abscisic acid stress were estimated by using IRGA CIRUS 3. Similarly, chlorophyll contents of transgenic and non-transgenic control cotton plants of drought and abscisic acid stressed plants were measured by following the same above procedure. The difference in photosynthetic rate and total chlorophyll contents was calculated in percentage before and after stressed condition.

### Analysis of morphological traits and fiber quality of AGL42_transgenic cotton lines

The T1 AGL42_ transgenic and non-transgenic cotton lines in field conditions were assessed for plant height and number of bolls. Total numbers of bolls in plants of each control and transgenic cotton lines were counted and the average number was statistically analyzed through One-way Anova. To observe any negative impact of AGL42 repressor protein on fiber traits, the fiber samples of all transgenic cotton plants as well as control plants of T_1_ generation were sent to CCRI (Central Cotton Research Institute), Multan for determination of fiber quality characteristics.

### Fluorescence in situ hybridization (FISH)

The best 35S:AGL42 transgenic cotton line was evaluated for transgene chromosomal location through Fluorescence in situ hybridization and karyotyping. The probe was labelled using commercial labelling kit (Mirus Bio LLC) to detect the transgene. FISH was performed on metaphase stage of chromosomes as described in by Noreen et al.^47^. Fluorescent signal was detected by DAPI and red63 dyes under fluorescent microscope Olympus (Model BX6l).

### Guidelines statement for Methodology

We assure that all methods were performed in accordance with the relevant guidelines and regulations.

## Supplementary Information

Supplementary Information.

## Data Availability

The datasets used and/or analyzed during the current study are available from the corresponding author on reasonable request.
