# Supplementary material for: Overexpression of the AGL42 gene in cotton delayed leaf senescence through downregulation of NAC transcription factors
Source: Sci Rep. 2022 Dec 6;12:21093. doi: 10.1038/s41598-022-25640-1 (PMC9727159; doi:10.1038/s41598-022-25640-1)
Supplement: Supplementary file 1 — Supplementary Information. [file 41598_2022_25640_MOESM1_ESM.pdf]

Supplementary Materials

Overexpression of the AGL42 gene in cotton delayed leaf senescence through downregulation of NAC transcription factors

Ayesha Latif[1], Saira Azam[1], Naila Shahid[1], Muhammad R. Javed[2], Zeshan Haider[2], Aneela Yasmeen[1], Sahar Sadaqat[1], Mohsin Shad[1], Tayyab Husnain[1], Abdul Q. Rao[1]

1. **Centre of Excellence in Molecular Biology, University of the Punjab, Lahore**
2. **Department of Bioinformatics and Biotechnology, Government College University Faisalabad, (GCUF), Allama Iqbal Road, Faisalabad-38000, Pakistan**

[*]Correspondence

Dr. Abdul Qayyum Rao
Professor, Centre of Excellence in Molecular Biology (CEMB), University of the Punjab, Lahore Pakistan
Email: qayyum.cemb@pu.edu.pk ; qayyumabdul77@yahoo.com,
Cell No. 0092 322 4676161

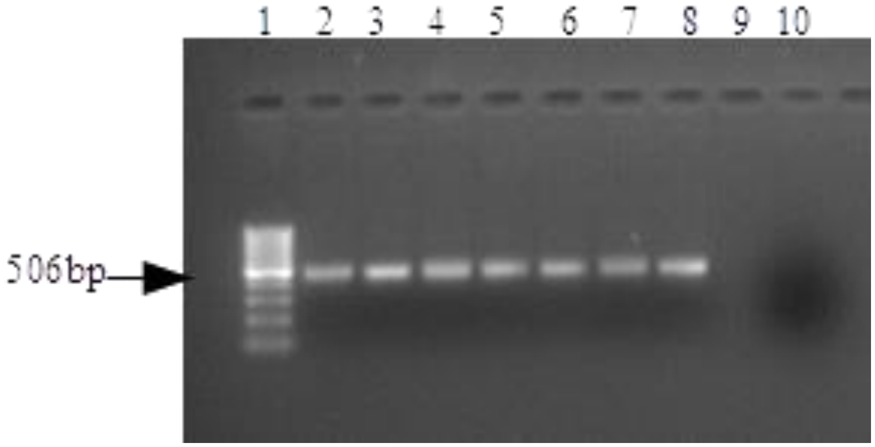

Figure S1: Confirmation of AGL42 gene in pCAMBIA 1301 vector through PCR

Lane 1: 100bp ladder

Lane 2: Positive control

Lane 3-8: Screened colonies

Lane 10: negative control

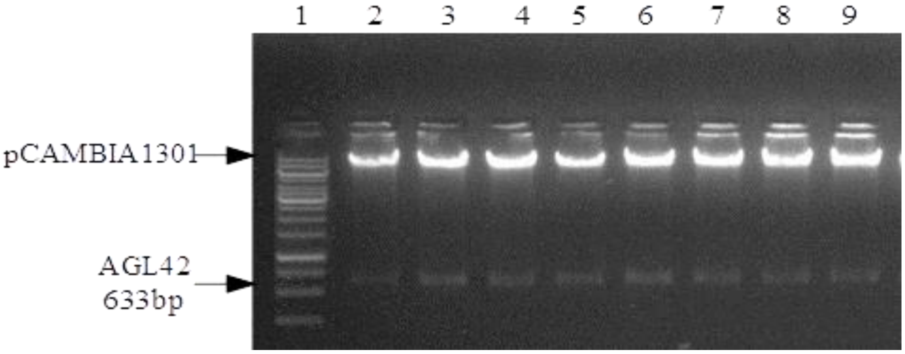

Figure S2: Digestion of AtAGL42 from pCAMBIA1301 through Nco1 and BglII enzymes
Lane 1: 1kb ladder

Lane 2-9: Digested positive clones

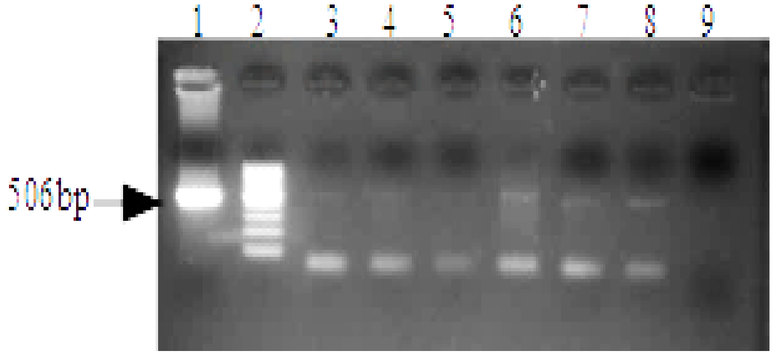

Figure S3: Confirmation of AGL42 gene in Agrobacterium through PCR Lane 1: Positive control Lane2: 100bp ladder Lane 3-8: Screened colonies Lane 9: Negative control

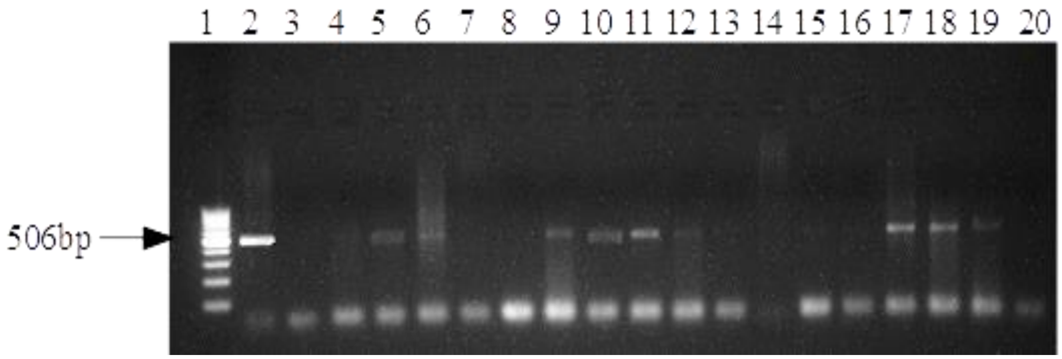

Figure S4: Confirmation of Agl42 gene in Putative transgenic cotton plants

Lane 1: 100bp ladder

Lane 2: Positive plasmid control

Lane 3-19: Putative transgenic plants

Lane 20: non-transgenic control plan

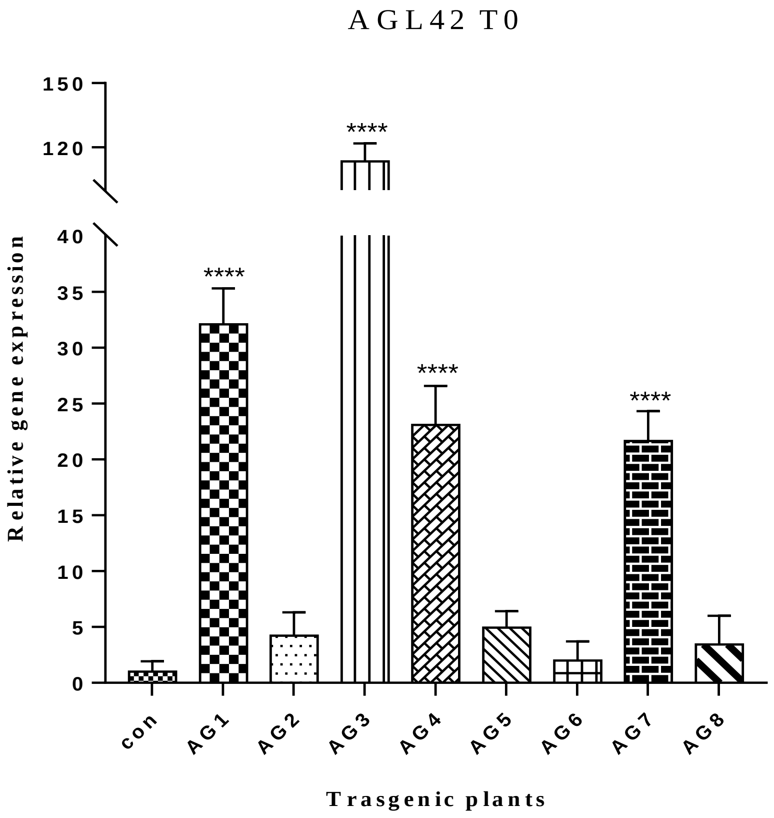

Figure S5: mRNA expression analysis of AtAgl42 gene in T0 generation through qRT-PCR.

Each bar depicts the average of technical triplicates from control and transgenic lines. Asterisks specify significant variation: “********P-value ≤ 0.0001”.

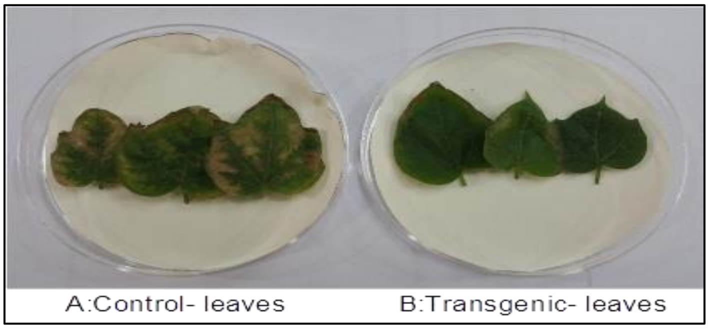

Figure S6: Leaf Detachment test after 7 days (A) Non transgenic line (B) Transgenic line

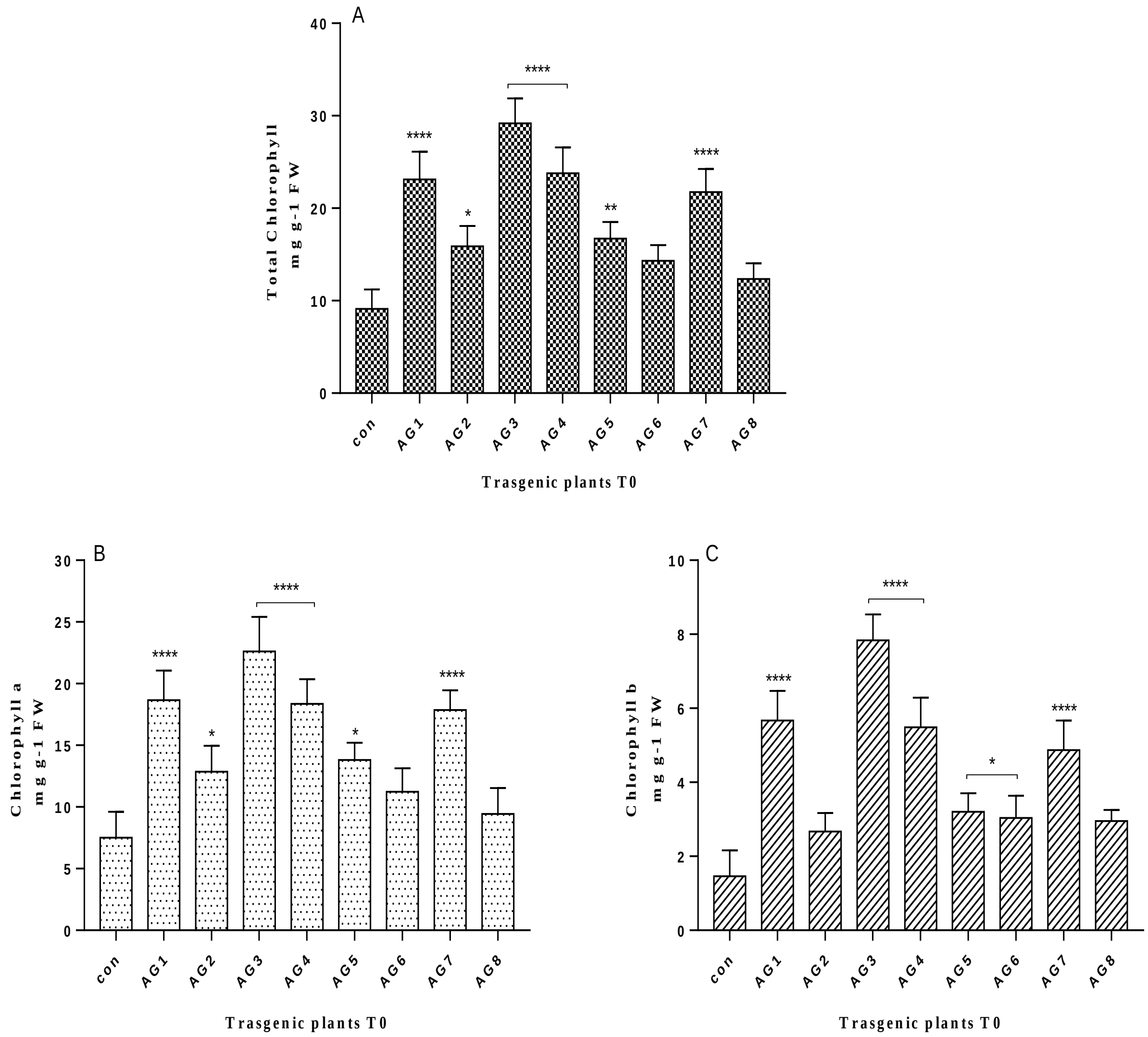

Figure S7: Chlorophyll contents in detached leaves of control and transgenic plants
A) Total chlorophyll; B) Chlorophyll a; C) Chlorophyll b. Each bar depicts the average of biological triplicates from control and transgenic lines. Asterisks specify significant variation: "********P value≤ 0.0001; *****P value ≤ 0.05".

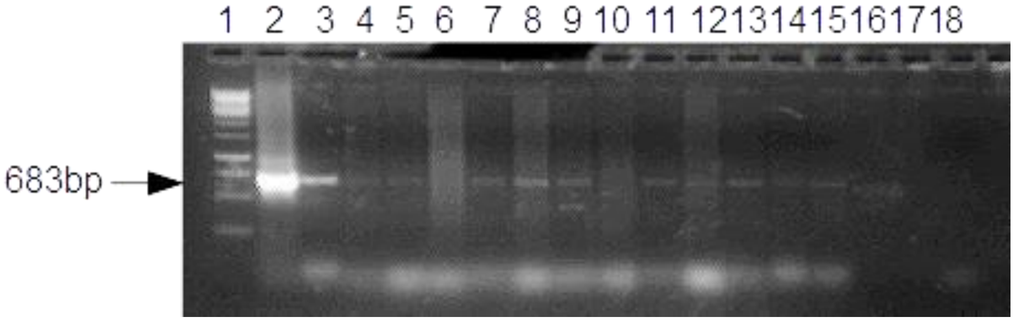

Figure S8: Confirmation of Agl42 gene in T1 transgenic cotton plants

Lane 1: 1kb ladder

Lane 2: Positive plasmid control

Lane 3-16: Transgenic plants

Lane 18: Non-transgenic control plant

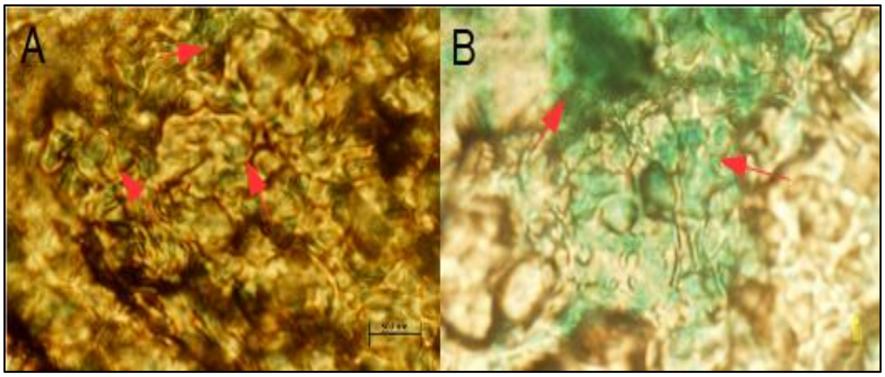

Figure S9: Microtomy sections indicating GUS: 35S_Agl42
(A) Leave section; (B) AZ (Flower bud) section

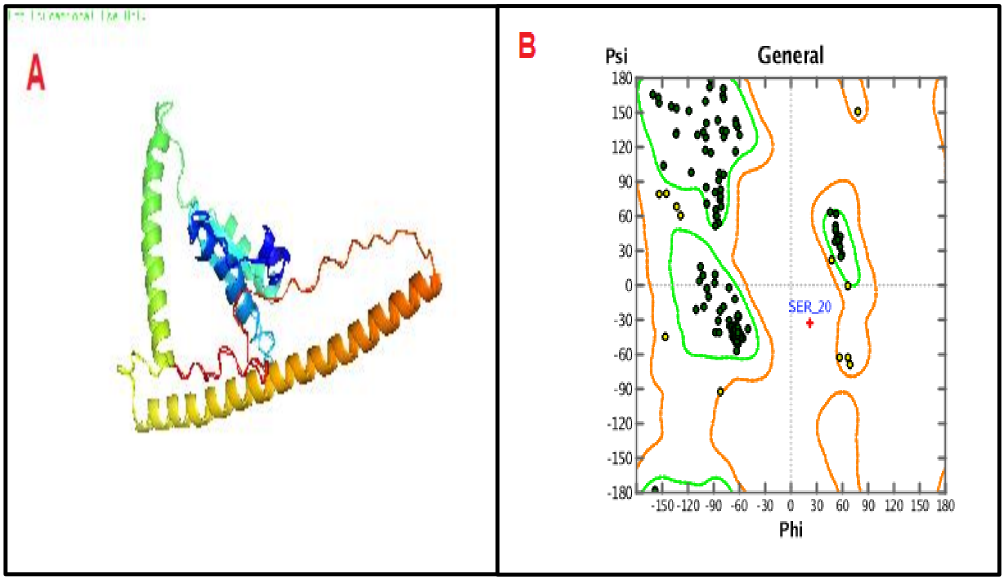

Figure S10: (A) 3D structure of transcription factor AGL42. (B) Ramachandran plot statistics analysis showing 92.7% residues in allowed region.

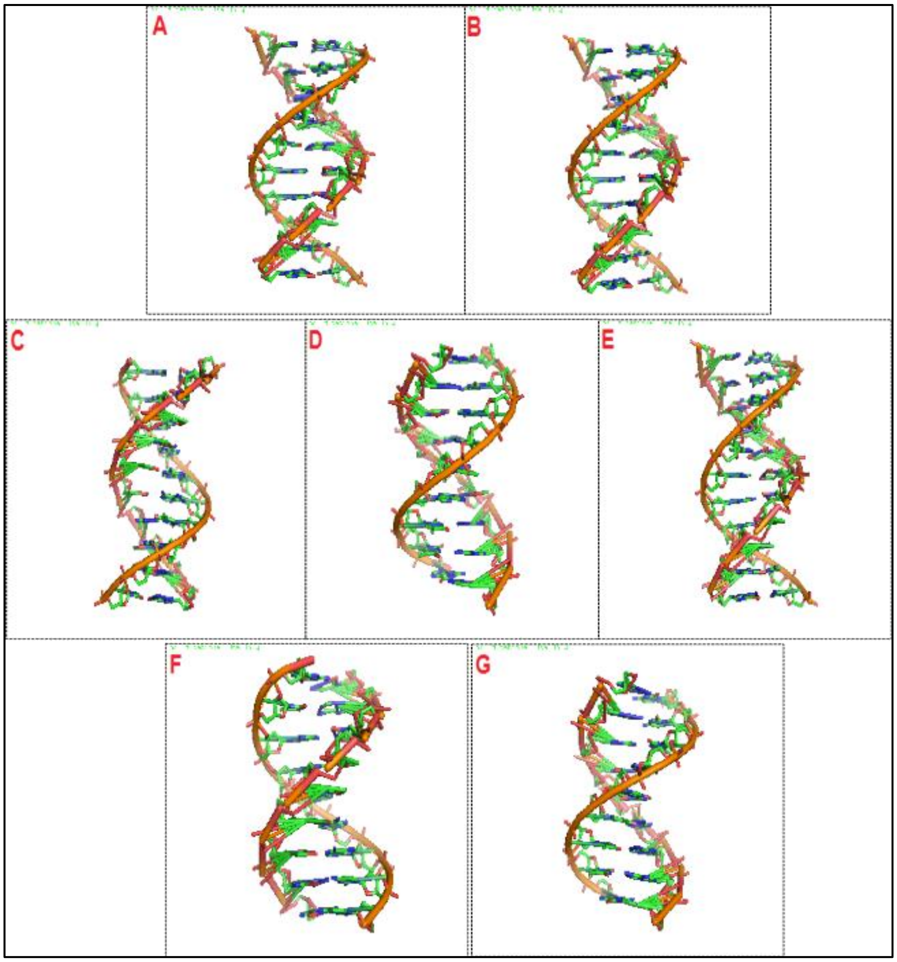

Figure S11: Modelling of DNA structures of transcription regulatory binding sites.
(A) 3D DNA structure of EDF1, (B) 3D DNA representation of EDF2, (C) 3D DNA model of NAC9 cotton promoter, (D)3D DNA structure of NAC12 cotton's promoter, (E) 3D DNA structure of NAC8 cotton's promoter, (F) DNA model of NAC14 cotton's promoter, (G) 3D DNA structure of NAC17 cotton's promoter.

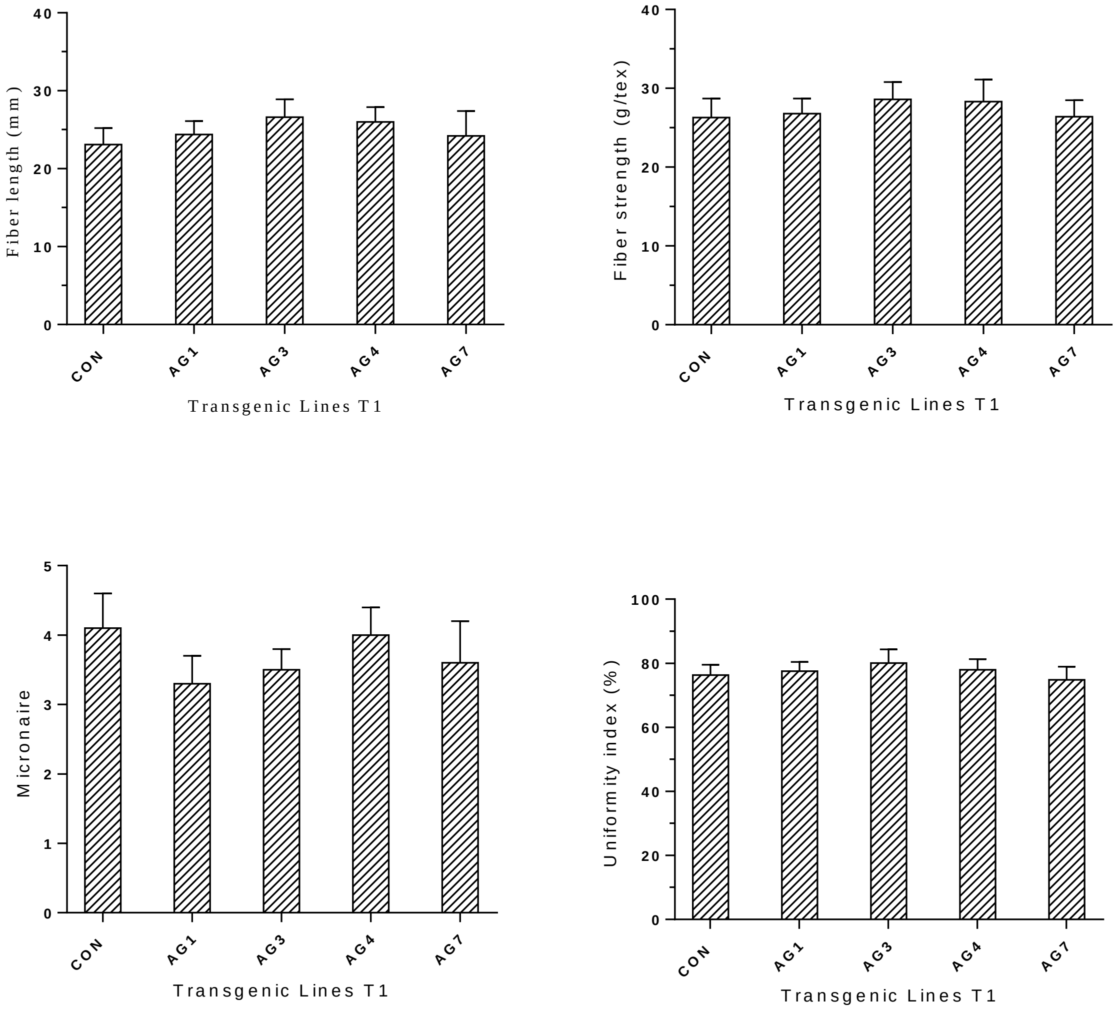

Figure S12: Fiber traits of transgenic and control plant lines.
(A) Fiber length: (B) Fiber strength: (C) Micronaire: (D) Uniformity index: All measurements signify the average of biological triplicate from each control and transgenic line.

Table SI: CArG sequence location in promoter of GhNAC genes

Gene ID-Promoters	Accession No	CArG sequence location
GhNAC8	JQ969023	-1091
GhNAC9	JQ969024	-111
GhNAC12	JQ969027	-230
GhNAC14	JQ969029	-1792
GhNAC17	JQ969032	-1010

Table SII: Primer sequences for PCR gene detection, real time and GhAct-4

Primer ID	Sequence (5´- 3´)	Annealing temperature	Product size
Agl42 (F-R)	5’ GGTCCGAGGAAAGATCGAAATG 3’	60°C	125bp
Agl42 (R-R)	5’ ACTGGGCATCACACAACACAGA 3’		
GhNAC8 (F-R)	5’TTGCCTCCTGGGTTTCGGTTC 3’	60°C	152bp
GhNAC8 (R-R)	5’ GTACCATTGCTTCTCTCCCATC 3’		(Shah *et al.*, 2013)
GhNAC9 (F-R)	5’ GAGATAGGAAGTACCCGAATG 3’	60°C	139bp
GhNAC9 (R-R)	5’ TTGAGTCCCTTTGGTGGCTTG 3’		(Shah *et al.*, 2013)
GhNAC11(F-R)	5’ GAGGACAAATAGGGCGACTGA 3’	60°C	154bp
GhNAC11(R-R)	5’ CTTGCCTTCCAGTCTGTATTC 3’		(Shah *et al.*, 2013)
GhNAC12 (F-R)	5’ AGGGGAAATCACTTGTTGGG 3’	60°C	162bp
GhNAC12 (R-R)	5’ GGGACTCTTTTGGAATACTC 3’		(Shah *et al.*, 2013)
GhNAC15 (F-R)	5’ TTTGCCTGGATTACCTGCTG 3’	60°C	163bp
GhNAC15 (R-R)	5’ CTTACTCCTGGCAACTTCTC 3’		(Shah *et al.*, 2013)
GhNAC16 (F-R)	5’ CCACGAGGAAACAAGACTGA 3’	60°C	126bp
GhNAC16 (R-R)	5’ TCTTCCTCCAATGCTGCTCT 3’		(Shah *et al.*, 2013)
NAC-14 (F-R)	5’ TCCAAGGAGGGATGCTGATGGT 3’	60°C	168bp
NAC-14 (R-R)	5’ AGGCGGAACTCAGGTGGTGTAT 3’		
NAC-17 (F-R)	5’AAGGAGGAGAGAACAGCACCAGG3’	60°C	148bp
NAC-17 (R-R)	5’TGGCTTTGATGGGCAGGTTTGG 3’		
GhACT4-F	5’TTGCAGACCGTATGAGCAAG 3’	60°C	105bp
GhACT4-R	5’ATCCTCCGATCCAGACACTG 3’		(Artico *et al.*, 2010)
AGL42-vec (F)	5’ TACAGTCTCAGAAGACCAAAGGGCAAT 3’	60°C	683 bp
AGL42-vec (R)	5’ TATCATATGGCTGGCCTCCTGCTT 3’		
Agl42 (F)	5’GTGTTGTGTGATGCCCAGTT3’	60°C	506bp
Agl42 (R)	5’GCCAATAAAAAGGTCCGTCTC3’		
